# 3,4,5-Tricaffeoylquinic acid induces adult neurogenesis and improves deficit of learning and memory in aging model senescence-accelerated prone 8 mice

**DOI:** 10.18632/aging.101748

**Published:** 2019-01-17

**Authors:** Kazunori Sasaki, Julie Davies, Noelia Geribaldi Doldán, Sayo Arao, Farhana Ferdousi, Francis G. Szele, Hiroko Isoda

**Affiliations:** ^1^ Alliance for Research on the Mediterranean and North Africa (ARENA), University of Tsukuba, Tsukuba City, Ibaraki 305-8572, Japan; ^2^ Interdisciplinary Research Center for Catalytic Chemistry, National Institute of Advanced Industrial Science and Technology (AIST), Tsukuba City, Ibaraki 305-8565, Japan; ^3^ Faculty of Pure and Applied Sciences, University of Tsukuba, Tsukuba City, Ibaraki 305-8571, Japan; ^4^ Department of Physiology, Anatomy and Genetics, University of Oxford, Oxford OX13QX, UK; ^5^ Faculty of Life and Environmental Sciences, University of Tsukuba, Tsukuba City, Ibaraki 305-8572, Japan; ^*^ Equal contribution

**Keywords:** TCQA, SAMP8, spatial learning and memory, neurogenesis, BMP signaling

## Abstract

Caffeoylquinic acid (CQA) is a natural polyphenol with evidence of antioxidant and neuroprotective effects and prevention of deficits in spatial learning and memory. We studied the cognitive-enhancing effect of 3,4,5-tricaffeoylquinic acid (TCQA) and explored its cellular and molecular mechanism in the senescence-accelerated mouse prone 8 (SAMP8) model of aging and Alzheimer’s disease as well as in human neural stem cells (hNSCs). Mice were fed with 5 mg/kg of TCQA for 30 days and were tested in the Morris water maze (MWM). Brain tissues were collected for immunohistochemical detection of bromodeoxyuridine (BrdU) to detect activated stem cells and newborn neurons. TCQA-treated SAMP8 exhibited significantly improved cognitive performance in MWM compared to water-treated SAMP8. TCQA-treated SAMP8 mice also had significantly higher numbers of BrdU+/glial fibrillary acidic protein (GFAP+) and BrdU+/Neuronal nuclei (NeuN+) cells in the dentate gyrus (DG) neurogenic niche compared with untreated SAMP8. In hNSCs, TCQA induced cell cycle arrest at G0/G1, actin cytoskeleton organization, chromatin remodeling, neuronal differentiation, and bone morphogenetic protein signaling. The neurogenesis promoting effect of TCQA in the DG of SAMP8 mice might explain the cognition-enhancing influence of TCQA observed in our study, and our hNSCs in aggregate suggest a therapeutic potential for TCQA in aging-associated diseases.

## INTRODUCTION

Aging and associated neurodegenerative conditions are a serious social problem. The United Nations report that the population aged > 60 years numbered 962 million in 2017 comprising 13% of the global population and is projected to be 2.1 billion in 2050. In addition, the number of the “oldest-old” (>80 years old) is expected nearly to triple by 2050 [1]. With a rapidly increasing aging population, the prevalence of age-related neurodegenerative disorders, such as Alzheimer’s disease (AD), is expected to rise dramatically in the next few decades. Yet, no current therapies effectively treat or cure AD. Amyloid beta (Aβ) aggregation forming senile plaques and accumulation of tau in neurofibrillary tangles characterize AD. These changes lead to neuronal and synaptic loss [2] as well as, excitotoxicity, oxidative stress and apoptosis [3]. Neuroinflammatory microglial activation is also important in the development of AD pathology [4]. All these pathophysiological mechanisms of AD are crucial for choosing an appropriate animal model. The senescence-accelerated mouse prone 8 (SAMP8) mice are considered as a good model for AD research as they exhibit inflammation, vascular impairment, gliosis, increased oxidative stress, Aβ accumulation, and tau hyperphosphorylation, accompanied by deterioration in memory and learning [5, 6]. A novel treatment approach of AD and other neurodegenerative diseases is to promote neuronal replacement in the affected tissue by stimulating neural stem cells (NSCs) and neural progenitor cells (NPCs) in the neurogenic niches.

Recent studies show that several natural polyphenols could promote neurogenesis and are preferable over currently used synthetic drugs for the treatment of neurodegenerative diseases as they are regarded as safe to use with fewer unwanted side effects. Caffeoylquinic acid (CQA) is one of the phenylpropanoids found abundantly in coffee beans, sweet potatoes, and propolis [7]. We have previously reported that CQAs has neuroprotective effects and activated ATP production due to its strong antioxidative effects in vitro and in vivo [8–10]. Our previous findings showed that administration of CQA-rich purple sweet potato extract could induce overexpression of proteins related to antioxidant, energy metabolism, and neuronal plasticity in the brains of SAMP8 mouse [10]. Furthermore, among CQA derivatives, 3,4,5-tricaffeoylquinic acid (TCQA) has the highest ability to increase ATP production in human neuroblastoma SH-SY5Y cells [9]. Taken together, we have hypothesized that TCQA might promote neurogenesis of NSCs and enhance neuronal plasticity due to its antioxidative and neuroprotective effects.

In the present study, we have performed in vivo experiments that postulate TCQA as a new natural therapeutic compound for the treatment and prevention of age-related diseases. We also have evaluated the effects of TCQA on fate determination of human NSCs (hNSCs), on oxidative stress, and in cell cycle regulation. Furthermore, we have performed a global gene expression profiling using DNA microarrays to elucidate the neurogenesis-promoting effects of TCQA.

## RESULTS

### TCQA improved spatial learning and memory of SAMP8 mice

**Figure 1:**
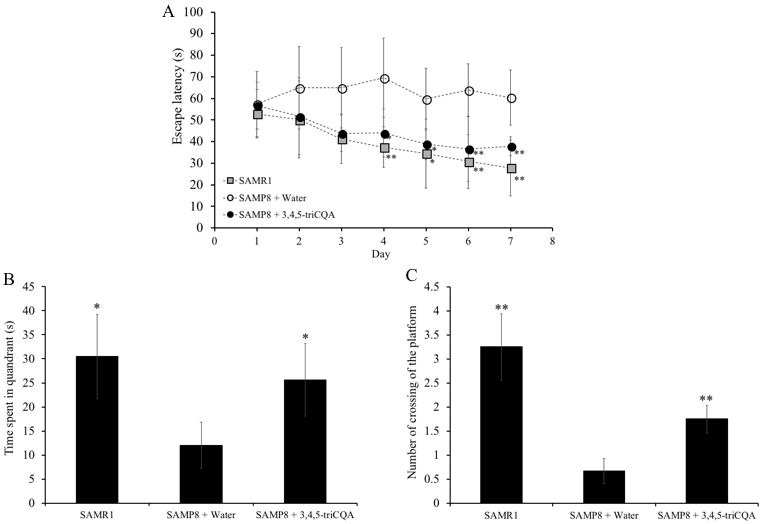
**Effect of ethanol extract of 3,4,5-triCaffeoylquinic acid (TCQA) on the spatial learning and memory as determined by escape latency of senescence-accelerated resistant mouse 1 (SAMR1) mice, senescence-accelerated prone mouse 8 (SAMP8) mice and SAMP8 TCQA-treated group determined by Morris water maze test (A).** Effect of ethanol extract of TCQA on the time spent in the target quadrant (**B**). Effect of ethanol extract of TCQA on numbers of crossings of platform by SAMR1 untreated and SAMP8 treated or untreated mice (**C**). * P < 0.05, ** P < 0.01 Compared with SAMP8 + water group.

To evaluate the effect of TCQA on spatial learning and memory in SAMP8 mice, the Morris water maze (MWM) test was employed. MWM is a widely used tool to assess spatial learning and memory in rodents. In the MWM, the animal is placed on a platform concealed under the surface of the water in the pool. The swimming time each mouse takes to swim to the platform indicates how quickly it can recall the location of the platform. To assess the effect of TCQA on spatial learning and memory, we measured the swimming time of the mice to reach the platform. As shown in Figure 1A, the escape latency time of TCQA-treated SAMP8 mice was significantly decreased compared to water-treated SAMP8 mice from 4^th^ to 7^th^ day training. On the other hand, there was no difference in the escape latency between SAMR1 mice and TCQA-treated SAMP8 mice.

Figure 1B and 1C show that the time spent in the target quadrant and the numbers of time the mouse crossed the platform were significantly lower in the water-treated SAMP8 group compared to both the TCQA-treated SAMP8 group and the water-treated SAMR1 group. However, no difference was observed in the occupancy and crossing of the target quadrant between the TCQA-treated SAMP8 group and the water-treated SAMR1 group.

### TCQA stimulates stem cells and increases neurogenesis in the dentate gyrus of SAMP8 mice

**Figure 2:**
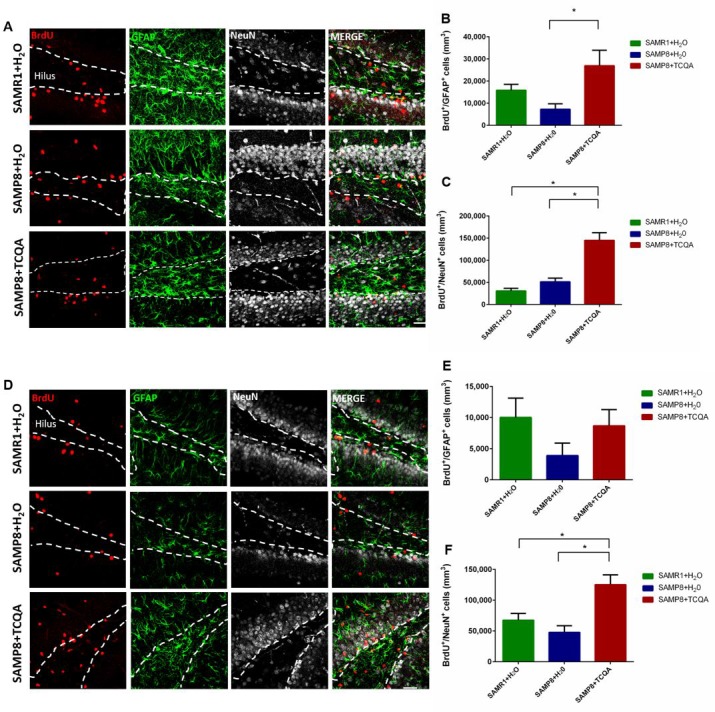
**Effect of oral administration of 3,4,5-triCaffeoylquinic acid (TCQA) on anterior (A–C) and posterior (D–F) DG stem cell activation and neurogenesis.** SAMP8 mice were administrated with TCQA (5 mg/kg) for 30 days. Photomicrographs show adult mouse brain in coronal sections containing the anterior (**A**) and posterior (**D**) DG processed for immunohistochemical detection of proliferating BrdU+ cells (red) and GFAP, a protein expressed by stem cells in the DG (green). Graphs represents the number of BrdU+ cells that co-express GFAP in anterior (**B**) and posterior (**E**) DG. Graphs represent the number of BrdU+ cells that co-express the mature neuronal marker NeuN in the anterior (**E**) and posterior (**F**) DG. Each bar represents the mean ±SEM * p < 0.05, ** p < 0.01 vs. SAMP8+TCQA group.

To study the effects of TCQA on NPC proliferation, we performed immunohistochemical detection of BrdU+ cells found in the dentate gyrus (DG) and the subventricular zone (SVZ) of SAMR1 mice and TCQA-treated and control SAMP8 mice. We also performed immunohistochemistry for the astrocytic marker GFAP and the neuronal marker NeuN. Cells that co-expressed BrdU and GFAP (BrdU+/GFAP+ cells) were identified as stem cells and cells that expressed BrdU+/NeuN+ were identified as newborn neurons. Both the numbers of BrdU+/GFAP+ cells and BrdU+/NeuN+ cells were significantly increased in TCQA-treated SAMP8 mice compared to untreated SAMP8 mice indicating that TCQA induced activation of NSC and increased the rate of neurogenesis in the anterior DG (Figure 2A–2C). In contrast, there was no significant change in the number of BrdU+/GFAP+ cells in the posterior DG (Figure 2E). TCQA treatment, however, did increase the number of BrdU+/NeuN+ cells in the posterior DG. These results suggest that increased hippocampal neurogenesis could be related to the improvement in learning and memory observed in MWM (Figure 1).

**Figure 3:**
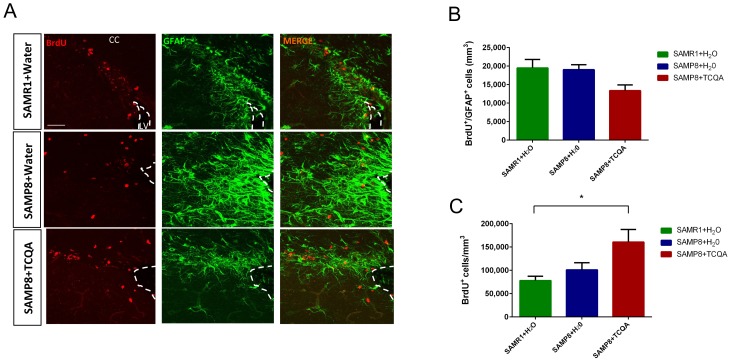
**Effect of oral administration of 3,4,5-tricaffeoylquinic acid (TCQA) on subventricular zone (SVZ) proliferation.** SAMP8 mice were administrated with TCQA (5 mg/kg) for 30 days (**A**) Photomicrograph shows adult mouse brain coronal sections containing the SVZ processed for immunohistochemical detection of proliferating BrdU^+^ cells (red) and GFAP+ (green), an astrocyte marker found in SVZ NSC. (**B**, **C**) Graphs represent the number of BrdU+/GFAP+ and BrdU^+^ cells, respectively in the different treatment groups.

The SVZ neurogenic niche is not involved in spatial memory and can exhibit different molecular regulation compared to the DG (Figure 3). Unlike the anterior DG, no significant difference was found in the number of BrdU+/GFAP+ cells in the SVZ among the three groups of mice (Figure 3B). However, the total number of BrdU+ cells in the SVZ was significantly increased in TCQA-treated SAMP8 mice compared to Water-treated SAMR1 mice (Figure 3C).

### TCQA increased cell viability of hNSCs

**Figure 4:**
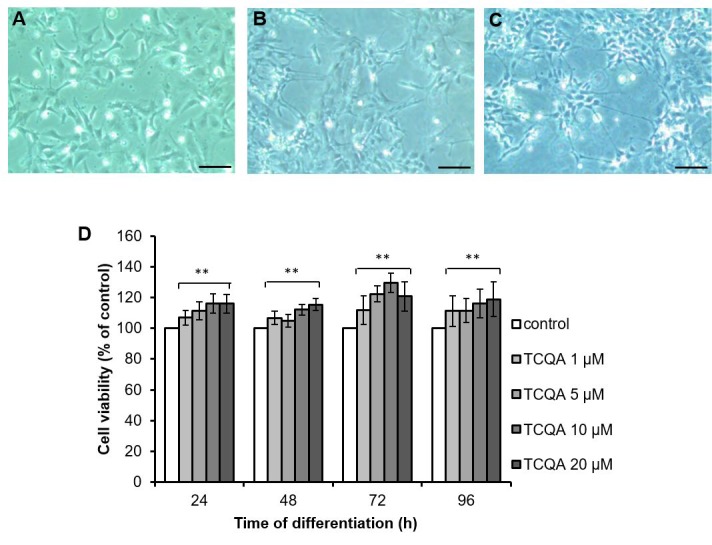
**The effect of 3,4,5-triCaffeoylquinic acid (TCQA) on cell viability of human neural stem cells (hNSCs).** hNSCs in undifferentiated state (**A**), induced differentiation for 96 h (**B**) and induced differentiation and treated with TCQA for 96 h (**C**). Time after differentiation, hNSCs were treated with TCQA (1, 5, 10, 20 μM) for 24, 48, 72, and 96 h (**D**). After the treatment, cell viability was measured by MTT assay. Data was set as % of control. Data were presented as mean ± SD. ** P < 0.01 Compared with control group.

To evaluate the cytotoxicity of TCQA on hNSCs, cell viability was examined using the 3-(4, 5-dimethylthiazol-2-yl)-2, 5-diphenyltetrazolium bromide (MTT) assay (Figure 4). The hNSCs were treated with increasing concentrations of TCQA (1, 5, 10, and 20 μM) in differentiation medium for 24, 48, 72, and 96 h. TCQA increased cell viability in a dose-dependent manner at all treatment times compared to corresponding controls (Figure 4). Cells treated with 20 μM of TCQA showed slightly less viability than the cells treated with 10 μM of TCQA at 72 h.

### TCQA promoted multilineage differentiation of hNSCs

**Figure 5:**
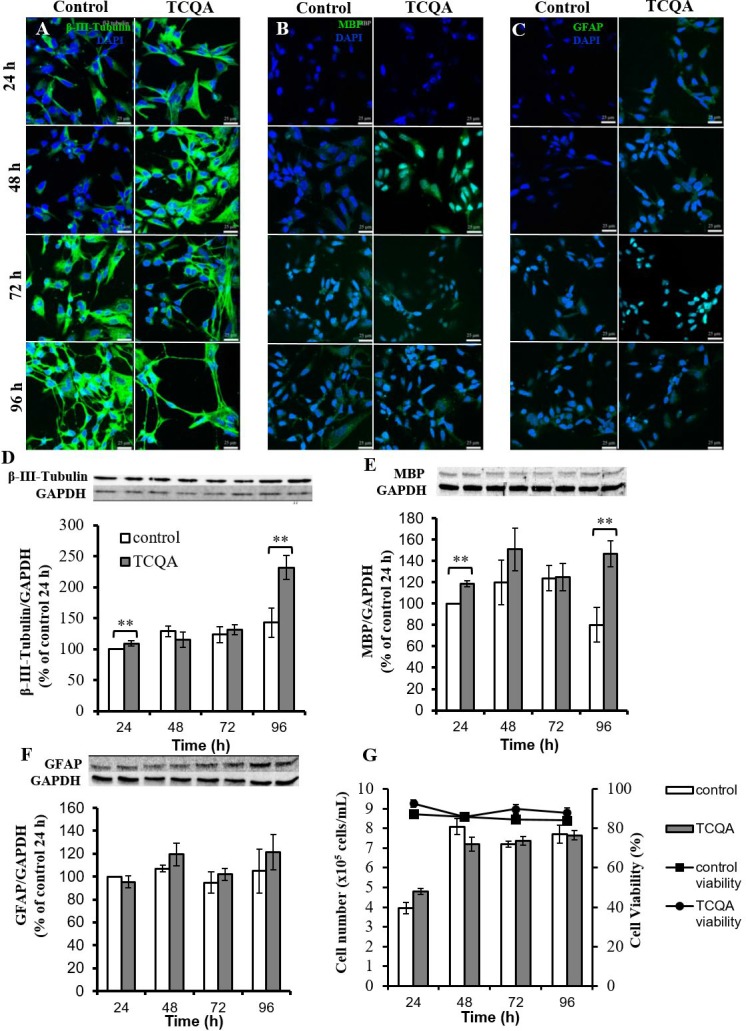
**The effect of 3,4,5-tricaffeoylquinic acid (TCQA) on fate, protein expression levels of differentiation markers, and cell proliferation of human neural stem cells (hNSCs).** Three differentiation markers (β3-tubulin: neuron, myelin basic protein (MBP): oligodendrocyte, glial fibrillary acidic protein (GFAP): astrocyte) were used. hNSCs were treated with differentiation medium with or without 10 μM TCQA. Expression levels of each differentiation marker were observed using confocal microscopy. Immunofluorescence images demonstrating the expression of β3-tubulin (**A**), MBP (**B**), and GFAP (**C**). hNSCs were treated with differentiation medium with or without 10 μM TCQA for 24 - 96 h. The expression level of each differentiation marker was determined by western blotting. Immunopositive bands of β3-tubulin (**D**), MBP (**E**), and GFAP (**F**) were quantified and expressed as a normalized value compared to glyceraldehyde-3-phosphate dehydrogenase (Gapdh). The cell number and cell viability were measured by ViaCount assay (**G**). * P < 0.05, ** P < 0.01 significance compared with control group.

It is known that hNSCs decide their fate within 24 h of induction of differentiation and differentiate into three kinds of cells; neuron, oligodendrocyte, and astrocyte, within three days [11]. We performed immunocytochemistry and western blotting using differentiation markers of three kinds of cells: β3-tubulin for neurons, MBP for oligodendrocytes and GFAP for astrocytes. Before immunocytochemistry and western blotting, we evaluated the cytotoxicity of TCQA (1–20 μM) on hNSCs by MTT assay. TCQA showed increased cell viability in a dose-dependent manner compared to corresponding control cells at 24h–96h (Figure 4). Immunocytochemistry showed that both control and TCQA-treated cells could differentiate into all lineages (Figure 5A–5C). However, TCQA substantially increased protein levels for all three differentiation markers. Compared to control cells, TCQA-treated cells showed significant increase in β3-tubulin expression at 24 h and 96 h of treatment (Figure 5D), and in MBP expression at 48 h and 96 h of treatment (Figure 5E) compared with those of in control cells. TCQA-treated cells also showed the increasing tendency in GFAP expression at 48 h and 96 h of treatment (Figure 5F). Glial differentiation takes place after neuronal differentiation. Furthermore, imaging by confocal laser scanning microscope suggested that TCQA treatment increased neurite length compared to the control condition (Figure 5A). It is known that β3-tubulin is expressed in both cell bodies and neurites (axons and dendrites) [12]. TCQA increased the expression of β3-tubulin in cell bodies at 24 h and 48 h. Then, both control and TCQA-treated cells extended neurites at 72 h. Finally, at 96 h, TCQA-treated cells showed longer neurites similar to axons. These results correspond with the protein expression by western blotting (Figure 5D). Both control and CQA-treated cells showed very low expression of MBP and GFAP compared to the expression of β3-tubulin (Figure 5B and 5C).

TCQA increased protein expression levels of all differentiation markers. There are two possibilities for this; first, TCQA promoted differentiation of hNSCs and second, TCQA promoted cell proliferation of hNSCs. To verify these two theories, we performed ViaCount assay to count cell number. hNSCs were treated with or without TCQA in differentiation medium for 24–96 h. In both untreated control and TCQA-treated groups, cell numbers were increased at 48 h compared to 24 h. However, no change was observed in the number of cells after 48h. There was also no significant difference in number of cells between control and TCQA-treated groups at any treatment time (Figure 5G). Additionally, cell viability was not changed at any treatment time in both control and TCQA-treated groups.

### TCQA induced G0/G1cell cycle arrest

**Figure 6:**
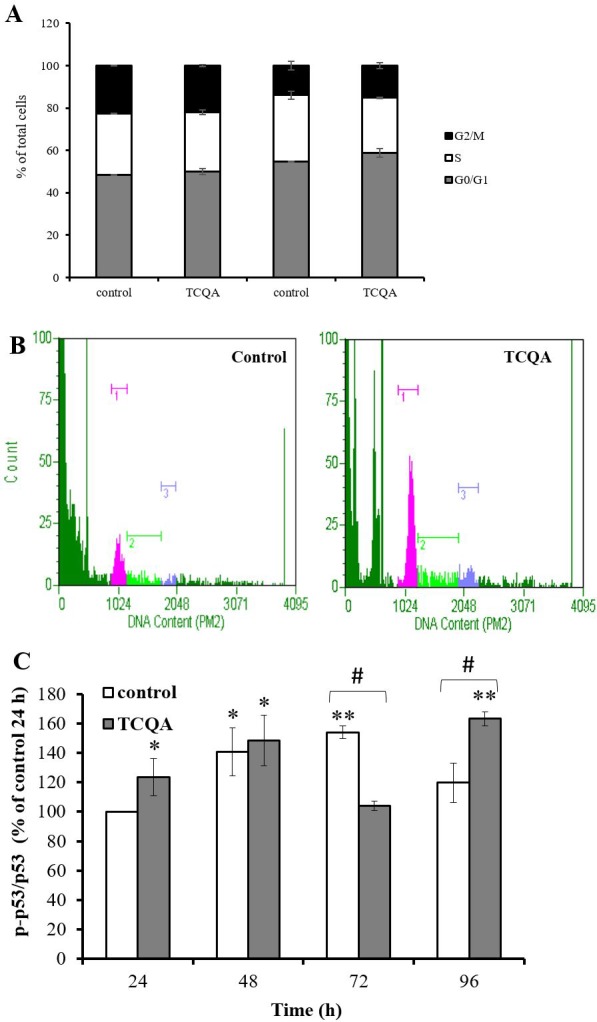
**The effect of 3,4,5-tricaffeoylquinic acid (TCQA) on cell cycle and phosphorylation of tumor protein p53 in human neural stem cells (hNSCs).** Cell cycle was determined by labeling cellular DNA. hNSCs were treated with growth medium or differentiation medium with or without 10 μM TCQA for 24 h. (**A**) Ratio of each cell cycle phase as a percentage of total cells. (**B**) The histograms show the cells in G0/G1 (pink peak on left), S (green center peak) and G2/M (blue peak on right). (**C**) hNSCs were treated with growth medium or differentiation medium with or without 10 μM TCQA for 24 - 96 h. Data was set as % of undifferentiated control. Data were presented as mean ± SD. * P < 0.05, ** P < 0.01 Compared with undifferentiated control cells. # P < 0.05 significance by student’s t test.

Cell cycle regulation is closely related to neurogenesis. It is well established that the G0 phase is related to neuronal differentiation [13]. Some G0/G1 cell cycle regulators, such as tumor suppressor protein p53 (p53), induce G0/G1cell cycle arrest and act as transcriptional factors modulating proneural gene expression. To investigate whether TCQA affects hNSCs cell cycle, we performed a cell cycle assay. hNSCs were treated with growth medium or differentiation medium with or without TCQA for 24 h. In undifferentiated hNSCs, TCQA treatment significantly increased the percentage of G0/G1 phase cells (P = 0.02) while S phase cells showed a significant decrease (P = 0.007). Differentiated hNSCs included more cells existing in the G0/G1 phase than the undifferentiated ones. Moreover, in differentiated hNSCs, TCQA increased the ratio of cells existing in G0/G1phase compared to control (Figure 6A and 6B). Next, we considered the expression of cell cycle regulator p53.

Phosphorylation of serine 15 in human p53 is an important regulator of G0/G1 cell cycle arrest. Phosphorylated p53 can activate p21, a cyclin-dependent kinase (CDK) inhibitor 1, or CDK-interacting protein 1 and p21 inhibits G1/S transition by binding directly to cyclin-CDK complexes. We measured phosphorylation of p53 by western blotting. Phosphorylation of p53 was significantly increased in TCQA-treated cells at 24, 48 and 96 h and decreased at 72 h compared to control cells (Figure 6C).

### TCQA affected intracellular Ca^2+^ levels, mitochondrial function, and intracellular reactive oxygen species (ROS) levels during early differentiation

**Figure 7:**
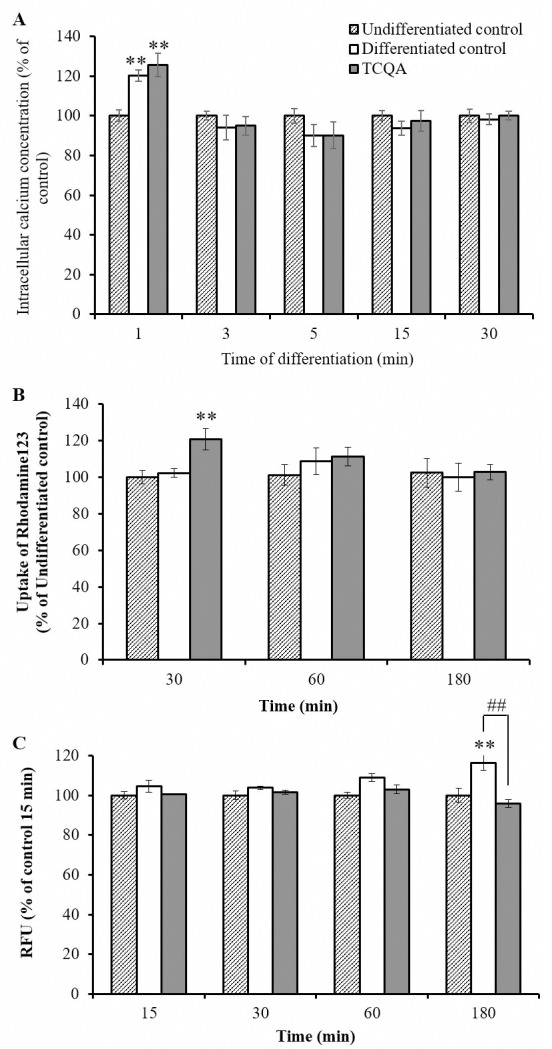
**The effect of 3,4,5-triCaffeoylquinic acid (TCQA) on intracellular Ca^2+^ levels, mitochondrial function, and intracellular reactive oxygen species (ROS) levels at very early phase of human neural stem cell (hNSCs) differentiation.** hNSCs were pre-treated with Fluo4 AM for 30 min followed by treatment with growth medium or differentiation medium with or without TCQA 10 μM for 1–30 min. Time after differentiation and TCQA treatment, intracellular Ca^2+^ level was detected by measurement of fluorescence intensity (**A**). hNSCs were treated with growth medium or differentiation medium with or without 10 μM TCQA for 30–180 min. TCQA was treated with rhodamine 123 and detected mitochondrial function by measuring the fluorescence intensity (**B**). Intracellular ROS levels were detected by measuring fluorescence intensity of DCF oxidized by ROS. hNSCs were pre-treated with DCFH-DA for 1 h followed by treatment with growth medium or differentiation medium with or without 10 μM TCQA for 15–180 min (**C**). Data was set as % of undifferentiated control. Data were presented as mean ± SD. * P < 0.01, ** P < 0.01 Compared with undifferentiated control cells.

Removal of growth factors initiates differentiation by inducing several early events. Firstly, Ca^2+^ is released from the endoplasmic reticulum into the cytoplasm in the absence of growth factors. Elevated Ca^2+^ levels in mitochondria increase oxidative phosphorylation to produce reactive oxygen species (ROS), which in turn activate signaling pathways related to neurogenesis. Thus, Ca^2+^ is considered a crucial initial factor of differentiation after removal of growth factors. We, therefore, investigated whether TCQA affects Ca^2+^ levels. After 1 min of TCQA treatment in undifferentiated cells, intracellular Ca^2+^ levels were significantly increased compared with undifferentiated controls (Figure 7A). TCQA-treated undifferentiated cells also showed slightly higher Ca^2+^ levels compared with undifferentiated control, but not higher than differentiation without TCQA, suggesting that it is the differentiation but not the TCQA that causes increased Ca^2+^. After 3 min of treatment, the intracellular Ca^2+^ levels were decreased in the TCQA treatment group compared with 1 min of TCQA treatment, suggesting that the elevated Ca^2+^ was transported to the mitochondria. After that, the decreased Ca^2+^ levels were gradually restored to baseline (Figure 7A).

After Ca^2+^ is transported to mitochondria, mitochondrial oxidative phosphorylation is activated. Mitochondrial oxidative phosphorylation uses proton concentration gradients between the inner and outer membranes of this organelle as driving power. The mitochondrial membrane potential (MMP) reflects this proton concentration gradient. Therefore, we investigated whether TCQA can affect mitochondrial oxidative phosphorylation by measuring the MMP. hNSCs were treated in a growth medium or a differentiation medium without (control) or with TCQA for 30–180 min. It was previously reported that removal of growth factors could activate mitochondrial oxidative phosphorylation in 30–180 min. After 30 min of the treatment, TCQA significantly increased MMP compared to both undifferentiated and differentiated controls. And, these increased MMP levels were restored to baseline after 60 and 180 min of TCQA treatment (Figure 7B).

TCQA is known to have high antioxidative activity, so there is a possibility that TCQA can reduce cellular oxidative stress by inducing differentiation. hNSCs were treated in a growth medium or a differentiation medium without (control) or with TCQA for 30–180 min. After 180 min of the treatment, TCQA significantly decreased intracellular ROS levels compared with differentiated controls (Figure 7C). However, there was no significant difference in ROS levels between undifferentiated and differentiated controls.

### TCQA affects gene expression during differentiation

**Figure 8:**
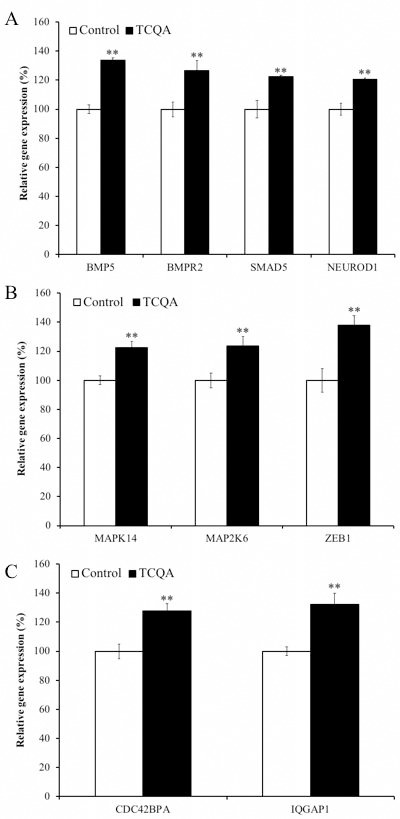
**The effect of 3,4,5-tricaffeoylquinic acid (TCQA) on gene expressions related to bone morphogenetic protein (BMP) signaling pathway.** Human neural stem cells (hNSCs) were treated with differentiation medium with or without 10 μM TCQA for 24 h. Genes expressing BMP ligand *BMP5*, BMP receptor 2 and *SMAD5* downstream BMP signaling pathway as well as the neuronal differentiation transcription factor *NEUROD1* were increased by TCQA (**A**). Genes related to p38–p53 signaling pathway regulating G0/G1 cell cycle arrest of hNSCs activated by the BMP signaling pathway were increased by TCQA (**B**). Genes related to the Cdc42 signaling pathway regulating neurite extension and activated by the BMP signaling pathway were increased by TCQA (**C**). Data was set as % of undifferentiated control. Data were presented as mean ± SD. ** P < 0.01 Compared with undifferentiated control.

We observed differences in protein expression of differentiation markers at 24h (Figure 8A–8C). We also found that hNSCs treated with TCQA could differentiate into more committed lineages than control NSCs. Numerous stemness-related genes are responsible for maintaining the undifferentiated state of NSCs. When differentiation is induced by growth factor withdrawal, decreased expression of stemness gene and increased expression of NSC fate-promoting genes can be observed. We, therefore, evaluated the effect of TCQA on global gene expression in hNSCs during differentiation at 24h. We repeated DNA microarray for three times and focused on genes whose expression was consistently altered in all three experiments. We classified genes into four functional groups highly relevant for neurogenesis: actin cytoskeleton (Supplementary Table 1), chromatin remodeling, neuronal development, and cell cycle-related genes (Table 1). Having dual functions, some genes were classified into multiple groups. TCQA increased bone morphogenetic protein (BMP) signaling pathway-related genes. TCQA increased expression of the *BMP5* ligand and the BMP receptor, type II (*BMPR2*) (Figure 8A). Furthermore, TCQA increased SMAD family member 5 (*SMAD5*) expression, which is activated by BMP signaling pathway and acts as a transcription factor during neuronal differentiation of hNSCs (Figure 6A) [14]. The expression of neuronal differentiation 1 (*NEUROD1*) is regulated by the BMP-SMAD signaling pathway and TCQA also increased *NEUROD1* expression (Figure 8A). TCQA also increased mitogen-activated protein kinase 14 (*MAPK14*) and mitogen-activated protein kinase kinase 6 (*MAP2K6*), which are responsible for coding, and for phosphorylation and activation of p38 respectively (Figure 8B). Activated p38 directly phosphorylates p53. Furthermore, activated p53 acts as a transcriptional factor and regulates zinc finger E-box bind 1(*ZEB1*). *ZEB1* is a transcription factor related to neuronal differentiation. We also found that

**Table 1:** Expression changes of cell cycle-, chromatin remodeling-, neuronal development-related genes regulated by 3,4,5-tricaffeoylquinic acid (TCQA)

Gene title	Gene symbol	Fold change	Function	Category
Ceramide Kinase Like	CERKL	1.51	cell cycle, activation of protein kinase C	Cell cycle
Translocated Promoter Region, Nuclear Basket Protein	TPR	1.45	negative regulation of mitosis, cell cycle checkpoint	
Structural Maintenance Of Chromosomes 4	SMC4	1.42	cell cycle, chromosome organization	
Remodeling And Spacing Factor 1	RSF1	1.35	cell cycle, histone binding,	
Dual Specificity Tyrosine Phosphorylation Regulated Kinase 2	DYRK2	1.32	activation of p53, cell proliferation, apoptosis, organization of the cytoskeleton and neurite outgrowth.	
Replication Timing Regulatory Factor 1	RIF1	1.29	postsynaptic density, actin projection, Required for checkpoint mediated arrest of cell cycle progression in response to DNA damage during S-phase (the intra-S-phase checkpoint)	
Plasminogen-related protein B	PLGLB	1.28	cell cycle arrest	
Transforming Acidic Coiled-Coil Containing Protein 3	TACC3	-1.22	cell cycle, cell proliferation in forebrain, protein transport, protein localization,	
Anaphase Promoting Complex Subunit 7	ANAPC7	-1.24	Cell cycle (M phase), protein modification,	
DNA Replication And Sister Chromatid Cohesion 1	DSCC1	-1.24	cell cycle (M phase), DNA replication	
Polo Like Kinase 4	PLK4	-1.24	cell cycle, regulation of centrosome duplication, protein kinase activity	
Minichromosome Maintenance Complex Component 4	MCM4	-1.26	DNA replication, Cell cycle	
HUS1 Checkpoint Clamp Component	HUS1	-1.30	Cell cycle, DNA repair, G1/S transition,	
SET domain containing (lysine methyltransferase) 8	SETD8	-1.31	Cell cycle (M phase), chromatin modification, negative regulation of gene and protein expression, methyl transferase	
alpha-thalassemia	ATRX	1.71	DNA repair, DNA methylation, zinc ion binding, forebrain development	Chromatin remodeling
Bromodomain Adjacent To Zinc Finger Domain 1A	BAZ1A	1.44	transcription, zinc ion binding, chromatin remodeling	
Lysine-specific demethylase 5A	KDM5A	1.37	chromatin organization, oxidation reduction, zinc ion binding	
Nuclear Receptor Corepressor 1	NCOR1	1.36	chromatin organization, negative regulation of transcription from RNA polymerase II promoter, negative regulation of JNK cascade, promotes histone deacetylation	
Bromodomain Adjacent To Zinc Finger Domain 1B	BAZ1B	1.31	chromatin organization, chromatin remodeling, chromatin regulation / acetylation	
Potassium Voltage-Gated Channel Subfamily Q Member 1	KCNQ1	1.30	chromatin regulation	
SKI proto-oncogene	SKI	1.30	negative regulation of transcription from RNA polymerase II promoter, negative regulation of activin receptor signaling pathway, TGF-beta signaling	
GLIS Family Zinc Finger 3	GLIS3	1.28	chromatin organization, zinc ion binding	
AT-rich interactive domain-containing protein 2	ARID2	1.28	Required for the stability of the SWI/SNF chromatin remodeling complex	
runt-related transcription factor 1; translocated to, 1 (cyclin D-related)	RUNX1T1	1.28	chromatin organization, zinc ion binding, binding to histone deacetylases and transcription factors	
G Protein Subunit Beta 4	GNB4	1.28	negative regulation of transcription from RNA polymerase II promoter	
Chromodomain Helicase DNA Binding Protein 4	CHD4	1.25	chromatin organization, SNF2-related, remodeling of chromatin by deacetylating histones	
Myb Like, SWIRM And MPN Domains 1	MYSM1	1.24	chromatin organization,	
Spen Family Transcriptional Repressor	SPEN	1.23	regulation of transcription from RNA polymerase II promoter, Notch signaling, negative regulation of transcription,	
Zinc Finger E-Box Binding Homeobox 1	ZEB1	1.43	negative regulation of transcription from RNA polymerase II promoter, zinc ion binding, Positively regulates neuronal differentiation.	Neuronal deveropment
Bone Morphogenetic Protein 5	BMP5	1.33	Hedgehog signaling pathway, TGF-beta signaling pathway,	
Bone morphogenetic protein receptor type 2	BMPR2	1.28	BMP signaling	
RAR Related Orphan Receptor A	RORA	1.27	neuron differentiation, cell differentiation in hindbrain,	
SMAD Family Member 5	SMAD5	1.22	BMP signaling	
Neurobeachin	NBEA	1.22	neuronal post-Golgi membrane traffic	
Leucine Rich Repeat Transmembrane Neuronal 1	LRRTM1	1.22	neuron projection, axon, growth cone, exhibits strong synaptogenic activity,	
Neuronal Differentiation 1	NEUROD1	1.20	neuron differentiation, cell cycle arrest	

TCQA increased activation of the cell division cycle 42 (Cdc42) signaling pathway that regulates actin cytoskeleton and neurite extension of hNSCs (Figure 8C).

## DISCUSSION

The present study sought to evaluate the effects of TCQA on spatial learning and memory and adult neurogenesis in SAMP8 mice as a model of AD and aging. It complemented these experiments with investigations into the molecular and cellular neurogenic effects of TCQA. The results obtained from the MWM illustrated that the TCQA-treated SAMP8 group had significantly increased spatial-learning acquisition and retention compared to both water-treated SAMP8 and SAMR1 control groups, and it, in fact, had similar levels to the SAMR1 control group. It is suggestive that TCQA may help to ameliorate, reverse or prevent the cognitive dysfunction that impairs learning and memory in SAMP8 mice. Moreover, the water-treated SAMP8 group remained in the platform quadrant less than SAMR1 and demonstrated a lower number of entries in the platform zone. In contrast, TCQA-treated SAMP8 mice spent more time in the platform quadrant and demonstrated higher number of entries in the platform zone compared to water-treated SAMP8 mice. Thus, three separate quantifications showed that the hippocampal dependant MWM task was worse in SAMP8 mice than in the SAMR1 controls and that TCQA "rescued" the defect.

SAMP8 mice are considered to have several advantages compared with other transgenic mice models in examining the effects of AD on neurogenesis. Several other transgenic mice models only show limited pathological hallmarks of AD. SAMP8 mice exhibit not only typical features of age-related cognitive impairment [15, 16] but also several pathological features of AD, including oxidative stress, Aβ alterations and tau phosphorylation [17–19]. We were especially interested in DG stem cells and neurogenesis since AD frequently commences in the hippocampus. Importantly, the number of activated DG NSC, as well as newborn neurons, were significantly increased in the anterior DG of TCQA-treated compared to water-treated SAMP8 mice. The anterior murine hippocampus has spatial memory functions whereas the posterior hippocampus has limbic functions [20]. Thus, it was interesting that the TCQA had different effects in these two regions; it activated both NSC and increased neurogenesis in the anterior DG but only increased neurogenesis in the posterior DG. Interestingly, TCQA stimulated proliferation in the SVZ but not NSC activation and future work will be needed to elucidate how these niches are differentially regulated by TCQA. TCQA induced hippocampal neurogenesis in both mouse adult NSC in vivo and human embryonic derived NSC cultures. Unfortunately, to the best of our knowledge, there are no reports about the pharmacokinetics of TCQA. However, Dan and colleagues reported that di-CQAs derivatives were absorbed after oral administration and the degradation products, mono-CQAs and caffeic acid (CA), were mostly found in tissues such as lung, kidney, brain, intestine, and small intestine, but not in plasma [21]. Therefore, we considered that after administration, TCQA was also degraded to mono-CQAs and caffeic acid, and mainly mono-CQAs and caffeic acid induced the neurogenesis. On the basis of these findings, we infer that TCQA-treatment improves spatial learning and memory through increased neurogenesis of hippocampal DG in SAMP8 mice.

In the previous study, we showed that 3,5-diCQA was found to improve spatial learning and memory in SAMP8 using MWM test [8]. Moreover, CQA derivatives have been reported to exert neuroprotective effects and promotion of energy metabolism through t overexpression of glycolysis-related [8], TCA cycle-related [10], and intracellular ATP production-related genes [8, 9]. Furthermore, in the previous result of metabolomics analysis, TCQA and 3,5-diCQA exhibited promotion of central metabolic pathways such as glycolysis and the TCA cycle [22]. Interestingly, TCQA showed the highest activity of promotion of ATP production and energy metabolism among other CQAs derivatives [9, 22]. Neurons have the high energetic demands for the maintenance and restoration of ion gradients dissipated by the repeated generation of postsynaptic potentials and action potentials, and the neurotransmitter cycle in the brain [23]. The previous report showed that neural stem and progenitor cells isolated from aged mouse forebrains display an aberrant metabolic phenotype characterized by decreased mitochondrial mass, lower oxygen consumption rates, and increased resistance to mitochondrial inhibitors, suggesting that mitochondrial function during aging contributes to impaired neurogenesis [23]. Therefore, we assume that TCQA, having the highest activity of promotion of energy metabolism, would also have the highest potential of the induction of adult neurogenesis compared with other CQA derivatives.

The central nervous system (CNS) has high energy demands high for nerve transduction, survival, proliferation, and differentiation. In this study, TCQA increased mitochondrial function and increased hNSC viability. NSCs, which are in the proliferative state, rely on glycolysis for their energy production [24]. The differentiation of NSCs gradually relies on oxidative phosphorylation, which is accompanied by the electron transport chain in mitochondria [25]. Therefore, enhanced mitochondrial activity is suggestive of NSC differentiation. As described above, NSCs can differentiate into three lineages: neurons, oligodendrocytes, and astrocytes. In this study, we found that TCQA-treated hNSCs expressed differentiation markers of all three lineages (β3-tubulin, MBP, and GFAP). We performed western blotting to measure differentiation markers and immunocytochemistry to evaluate neurite outgrowth, which is the main characteristic of differentiating cells [26]. Our results showed that TCQA-treated hNSCs eventually increased differentiation markers compared with control cells, especially β3-tubulin expressed primarily in young neurons. Our results also showed that TCQA-treated hNSCs transiently increased GFAP and MBP expression. GFAP is widely used as an astrocyte marker [27] and is also expressed in NPCs. In contrast, MBP is widely accepted as a marker of adult oligodendrocytes [28]. Altogether, our results suggested that TCQA promoted differentiation of hNSCs at least toward the neuronal lineage and suggests the possibility of using TCQA to promote neurogenesis. However, the mechanism resulting in this effect of TCQA is still unclear. Therefore, we also evaluated whether TCQA affects early events in differentiation and gene expression.

Because cell cycle regulation is involved in a large array of intracellular differentiation events, we also studied the effects of TCQA on cell cycle regulation. TCQA-treated cells increased the ratio of cells in the G0/G1 phase in both undifferentiated and differentiated cells. It is widely known that G0/G1 cell cycle arrest is related to differentiation, as G0/G1 cell cycle progression factors negatively regulate basic helix-loop-helix, a protein structural motif that characterizes a family of transcription factors, responsible for neurogenesis [29]. In addition, the phosphorylation of p53, an important regulatory factor of G0/G1 arrest, was increased by TCQA-treatment. Our microarray results showed that several genes related to cell cycle were affected by TCQA-treatment. *DYRK2* acts as an activator of p53 and negative regulator of G1/S transition [30]. *RIF1* acts in the checkpoint of G1/S and increases G0/G1 arrest [31]. TCQA-treated cells increased both *DYRK* and *RIF1* up to 1.32 and 1.29 respectively, suggesting the suppression of G1/S transition and the increase of G0/G1 arrest. *SETD8* and *MCM4* begin to be expressed at S phase and reach expression peaks at G2/M phase, thus, the downregulation of *SETD8* and *MCM4* to -1.31 and -1.26 respectively, suggests the ratio of cells in S-G2/M phase was decreased. Similarly, *HUS1* acts during S phase as a DNA damage checkpoint [32] and *PLK4* promotes DNA replication through duplicating centrosomes [13]. *DSCC1* is a component of the alternative replication factor complex (RFC) and loads *PCNA* on DNA [33, 34]. The decrease in expression of these genes (*HUS1*: -1.30, *PLK4*: -1.24, *DSCC1*: -1.24, respectively) related to DNA replication is in concert with the finding that the proportion of cells in S phase being decreased by TCQA. *ANAPC7* (-1.24) is a component of anaphase-promoting complex/cyclosome and controls G1 phase progression [35]. *TACC3* (-1.22) is suppressed by the activated p38-p53-p21 signaling pathway and induces G0/G1 arrest [36]. In this study, p53 was activated and gene expression of *MAP2K6* (1.23), which phosphorylates p38 protein, and *MAPK14* (1.22), which activates the JNK signaling pathway, was increased by TCQA treatment. From these results, it is assumed that TCQA increased G0/G1 arrest by negatively regulating G1/S transition via changing expressions of various genes and moving hNSCs toward more lineage-committed cells. It is important to note that modulating cell cycle phase lengths can regulate rates of neurogenesis *in vivo* in the cerebral cortex and the dentate gyrus stem cell niche [37, 38]. Thus, it will be fascinating to dissect with functional studies which of the genes above are necessary for TCQA's effects on neurogenesis. Actually, our microarray result showed that the fold change of gene expression was usually below 1.5-fold. Therefore, our microarray analysis showed that the number of genes left after performing fold change cut off more than 1.5 or 2.0 were small. It was suggested that biologically fewer genes show a drastic change, therefore, using the stringent criteria for differentially expressed genes may lead to overlooking some important biological functions. Thus, to understand the integrative response of cells to TCQA treatment, we chose to evaluate the genes that did not vary widely in expression but had important biological significance [39].

A series of events occurred in response to the removal of growth factors from NSCs to induce differentiation. Intracellular Ca^2+^ levels were increased immediately after the removal of growth factors. Then after 3 min of removal of the growth factors, the Ca^2+^ levels were decreased in both control cells and TCQA-treated cells (Figure 7A). This result suggested that Ca^2+^ was released from the endoplasmic reticulum (ER), was transported to the mitochondrial matrix through Ca^2+^ transporters located at the ER-mitochondria contact site to the cytosol [40], and consequently was transferred to mitochondria. In addition, TCQA-treated cells increased MMP at 30 min compared to differentiated control cells. Activated mitochondria produce ROS and energy by mitochondrial oxidative phosphorylation. ROS can induce signal transduction related to neurogenesis under normal conditions. However, overproduction of ROS leads to mitochondrial DNA damage and eventually negatively affects neurogenesis [41]. The production of ROS was significantly decreased by the induction of differentiation in TCQA-treated cells compared to differentiated cells. However, no significant difference was observed in production between TCQA-treated and undifferentiated cells.

Importantly, TCQA-treated cells exhibited increased expression of several genes related to neuronal development. Neurobeachin is important for the formation and composition of central synapses and is also essential for neuromuscular synaptic transmission [42, 43]. *ZEB1* acts as a neuronal transcription factor and positively regulates neuronal differentiation [44]. Our results showed that TCQA also increased BMP signaling pathway-related genes. As well as the actin cytoskeleton, the BMP signaling pathway has strong effects on neural fate commitment. BMP signaling initially promotes neurogenesis by pushing progenitors towards the neuronal fate and suppressing the oligodendroglial fate [11]. In addition, *SMAD5* is a transcription factor, which is a pivotal intracellular effector of the BMP signaling pathway. *SMAD5* is activated by BMP-binding the BMP receptor. Furthermore, BMP signaling induces expression of neuron-specific β3-tubulin. Besides, TCQA-treated cells also exhibited increased expression of *NEUROD1*, which acts as a transcriptional factor for neuronal differentiation genes under activated BMP-SMAD signaling. These results suggested that TCQA might promote neuronal differentiation through the BMP signaling pathway.

Axonal transport is another important cellular process of neurons that transports mitochondria, lipids, synaptic vesicles, proteins, and other cell components between the cell body and axons. Axonal transport occurs throughout life in neurons for their survival. Kinesin and dynein are important motor proteins, which move cargoes anterogradely (from the cell body to axon tip) and retrogradely (from axon to cell body), respectively [45]. Kinectin 1 (*KTN1*), a kinesin receptor, and kinesin family member protein 21A (*KIF21A*), one of the motor proteins of kinesin, were increased by TCQA treatment (Supplementary Table 1). Furthermore, the expression of dynein cytoplasmic 1 light intermediate chain 2 (*DYNC1LI2*) and dynein cytoplasmic 1 heavy chain 1 (*DYNC1H1*) were also increased in TCQA-treated cells compared to non-treated cells (Supplementary Table 1). The increased expression of these genes suggests that the elongated neurites might have neuronal functions. Current evidence suggest that deficits in axonal transport contribute to the pathogenesis of several neurodegenerative disorders [46].

## CONCLUSION

The development of new strategies to promote neurogenesis in aging-related diseases is a major therapeutic challenge. Our results suggest that TCQA-treatment improves spatial learning and memory through the increase of neurogenesis of hippocampal DG. Importantly we showed that TCQA also has pro-neurogenic effects in human cells, that may occur via activation of the BMP signaling pathway. We, therefore, postulate that TCQA could be a new agent capable of increasing NPC proliferation in the DG and improve learning and spatial memory. Therefore, it will be important to determine how functionally mature and long-lived the new neurons are and if they are directly responsible for various aspects of memory. It may also be possible to modify TCQA to create an optimal new therapeutic drug for the treatment of aging-associated neurodegenerative diseases.

## MATERIALS AND METHODS

### TCQA preparation

We used TCQA for our study as it promotes the highest neuronal ATP production compared with other CQA derivatives [9]. As the extraction and purification/isolation of a large amount of TCQA from plant materials / natural biological sources is difficult, we used synthesized TCQA. Dr. Kozo Sato from Synthetic Organic Chemistry Laboratories, the FUJIFILM Corporation (Kanagawa, Japan) provided us with the synthesized TCQA with 97% purity. TCQA was dissolved in 70% EtOH (TCA stock), and then, for *in vitro* assays, TCA stock was dissolved in the medium. For *in vivo* assays, TCA stock was dissolved in drinking water.

### Animal subjects, TCQA administration and Bromodeoxyuridine (BrdU) labeling

Four-month-old adult male SAMP8 and SAMR1 (purchased from the Japan SLC company, Japan) mice were used for the experiment. All mice were allowed to acclimate to the laboratory conditions for seven days under controlled conditions of temperature (21–23ºC) and light (light: dark 12:12) with free access to food and water. SAMP8 mice were randomly divided into two groups, SAMP8 control group (n = 6) and TCQA-supplemented group (n = 7). SAMR1 mice (n = 10) were used as normal aging controls. TCQA (5 mg/kg) was mixed with drinking water and then was directly administered by oral gavage with a feeding tube and a syringe every day for 30 days. BrdU (1mg/ml) was diluted in drinking water and was provided to the animals for nine consecutive days starting from the 14^th^ day of TCQA treatment. The schedule of BrdU administration followed by washout and sacrifice was chosen, as it is capable of detecting differences in stem cell activation as well as neurogenesis. All animal procedures were approved by the Animal Study Committee of University of Tsukuba (No.16–042) and were handled according to the guidelines for the Care and Use of Animals approved by the Council of the Physiological Society of Japan.

### Morris Water Maze (MWM)

MWM was carried out using a circular pool (120 cm in diameter and 45 cm in height) with a featureless inner surface. The pool was filled to a depth of 30 cm with water (23 ± 2°C) and was divided into four quadrants. A platform (10 cm in diameter) was placed in the northeast quadrant and was submerged 1 cm below the water surface so that it was invisible at water level. The mice were then given four trial sessions each day for one week on a platform fixed in the same location for the whole duration of the experiment. Each mouse was placed on the platform for 15 s every day for 7 days.

After 7 days of trials, the hidden platform was removed from the pool and the mice were then placed in the opposite location of the platform and were allowed to swim for 60 s. The number of crossings over the previous position of the platform and the time spent in the target quadrant in which the platform was hidden during the acquisition trials were recorded as measures for spatial memory.

### Tissue processing and immunostaining

At the end of MWM, animals were sacrificed and brains were removed. Ten brains of SAMR1 mice, six brains of SAMP8 control mice and seven brains of TCQA supplemented SAMP8 mice were harvested. The brains were fixed in 4% paraformaldehyde (PFA) for 24 h and then cryoprotected in 30% sucrose (w/v) in phosphate buffered saline (PBS) for 48 h, both at 4 ºC. Serial 30 µm coronal brain sections were obtained using a microtome on dry ice. Sections were stored at -20ºC in cryoprotectant solution (ethylene glycol, glycerol, 0.1 M phosphate buffer, pH 7.4, 1:1:1:2 by volume). Sections were washed in 0.1 M PBS 3 × 10 min and then blocked with 50 mM glycine in PBS to reduce autofluorescence of PFA-fixed tissue. For immunohistological detection of BrdU, sections were incubated in HCL (2N) at 37ºC for 1 h before blocking. Sections were washed in PBS, before blocking for 1 h in PBS containing 0.1% Triton X-100 and 10% Donkey Serum (DS, Sigma). Primary antibodies used were anti-BrdU (1:400, sheep, Abcam Ab1893), anti-GFAP (1:500, rat, Invitrogen, AF568), anti-NeuN (1:400, mouse, Millipore MAB377). The secondary antibodies were conjugated to Alexa-488, -568 or -647 (Invitrogen Paisley, Renfrewshire, UK; 1:500).

### Culture of hNSCs

The hNSCs derived from H9 hNSCs were purchased from Gibco, Japan. The hNSCs were cultured in 60 mm Petri dishes (BD Falcon) or in 6-well plates (BD Falcon) with a growth medium Knock Out^TM^:1:1 (v/v) mixture of Dulbecco’s minimum essential medium (Sigma, U.S.A.) and Ham’s F-12 nutrient mixture (Sigma, U.S.A.) supplemented with 2% StemPro neural supplement, 20 ng/mL of fibroblast growth factor (FGF) basic recombinant human, 20 ng/mL epidermal growth factor (EGF) recombinant human and 2 mM GlutaMAX-I supplement. For adhesion, 60-mm Petri dish or 6-well plate coated with CELLStart were used. The hNSCs were cultured at 37ºC in a 95% air / 5% CO_2_ humidified incubator. The medium was changed every two days to keep cells undifferentiated.

### Determination of cell viability

Cell viability was measured using the MTT assay. hNSCs were treated with 5–30 μM of TCQA for 72 h. After TCQA treatment, 5 mg/ml of MTT was added and the cells were incubated for further 12 h. The MTT formazan was dissolved in 100 μl of 10 % SDS (w/v). Absorbance (570 nm) was measured using a microtiter plate reader (Dainippon Sumitomo Pharma Co., Ltd., Japan).

### Differentiation assay and immunocytochemistry

To induce differentiation, hNSCs were seeded into CELLStart coated culture vessels at the density of 2.5 x 10^4^ cells/cm^2^. After 48 h, growth medium was replaced by differentiation medium without growth factors: KnockOut DMEM/F12 supplemented with 2% StemPro neural supplement and 2 mM GlutaMAX-I supplement. The medium was changed every 2 to 3 days. For immunocytochemistry, cells were seeded in the Nunc Lab-Tek Chamber Slide System (Thermo Scientific, Japan) at the same density and were incubated at 37ºC for 48 h. After incubation, cells were treated with differentiation medium with or without TCQA at 37ºC for 24–96 h. After treatment, cells were washed with PBS and were fixed with 4% (w/v) PFA diluted in PBS for 30 minutes at room temperature. The fixed cells were washed with PBS 3 times for 5 minutes. After washing, cells were blocked overnight at 4ºC with saturation solution of 5% Fetal Goat Serum and 0.1% TritonX-100 (Sigma, Japan) in PBS. Blocking buffer was removed after incubation and primary antibodies (Novex Goat anti-Rabbit IgG (H+L), Sigma, Japan) were added in a specific antibody dilution buffer (1% Bovine Serum Albumin and 0.3% TritonX-100 in PBS). After the incubation with the primary antibody overnight at 4ºC, cells were washed with PBS and incubated with the secondary antibody (Alexa Flour 488 conjugate goat anti-rabbit IgG (H+L) (1:1000), Thermo SCIENTIFIC, Japan) for 1–2 h at room temperature avoiding light exposure. After incubation, cells were washed with PBS and nuclei were counterstained with DAPI using drops of ProLong Gold Antifade Mountant (Thermo Scientific, Japan). Fluorescence was detected with Leica DMI 4000B (Leica, Germany).

### Protein extraction

The hNSCs were seeded in 60 mm Petri dish (BD Falcon) at the density of 2.5×10^4^ cells/cm^2^ and were incubated at 37ºC for 48 h. After the incubation, cells were treated with differentiation medium with or without 10 μM TCQA for 24–96 h at 37ºC. After the treatment, cells were washed with cold PBS twice and then 100 μl of RIPA buffer (Sigma, Japan) including 1% protease inhibitor (Sigma, Japan) was added. Cells were incubated for 5 min on ice and cell lysate was collected in Cryo-tubes and was frozen in liquid nitrogen for 15 min. The frozen solution was melted on the ice and centrifuged (4ºC, 10,000g, 15 min) in 1.5 ml Protein LoBind Tube (Eppendorf, Japan). The supernatants were transferred into new tubes and protein concentrations were quantified with 2-D Quant kit (GE Healthcare, Japan). Protein samples were stored at -80ºC until use.

### Western blotting

Protein samples (20 µg) were separated using 10% SDS-PAGE and were transferred to a polyvinylidene difluoride membrane (PVDF, Merck Millipore, USA). Western blot was performed as previously described (Matsukawa et al., 2017). Primary antibodies used were anti-β-III-Tubulin (CST, Japan); anti-myelin basic protein (MBP) (Sigma, Japan); anti-glial fibrillary acidic protein (GFAP) (Sigma, Japan); anti-glyceraldehyde 3-phosphate dehydrogenase (GAPDH) (1:200, Santa Cruz, Japan); rabbit anti-Phospho-p53 (S15, CST, Japan) and rabbit anti-p53 (1:1000, CST, Japan). Secondary antibodies were IRDye 800CW donkey anti-rabbit IgG (1:1000) or IRDye 680LT goat anti-mouse (1:20000) from LI-COR, Inc., Lincoln, NE, USA. The signal was detected using the Odyssey FC Imaging System (LI-COR, Inc, NE, USA).

### Cell cycle assay

Guava Cell Cycle Reagent (Millipore, Japan) was used to evaluate the effects of TCQA on cell cycle regulation in undifferentiated and differentiated cells. The hNSCs were cultured in 60 mm Petri dishes at a density of 2.5 × 10^4^ cells/cm^2^ and were incubated at 37ºC for 48 h. Cells were treated with or without TCQA (10 µM) in growth or differentiation medium at 37ºC for 24 h. After incubation, cells were washed and treated with StemPro accutase to detach from the dish and were centrifuged twice at 200 g for 4 min. One ml of cold 70% ethanol was next added and cells vortexed. Cells were fixed at 4 ºC for 1–12 h. Then the cells were centrifuged twice at 450 g for 5 min. After the last centrifugation, Guava Cell Cycle Reagent (Guava Technologies, Japan) was added to cells and was incubated for 30 min at room temperature in the darkness. After incubation, cell cycle was determined using Guava easyCyte (Millipore, Japan).

### Measurement of intracellular Ca^2+^ levels in hNSCs

After inducing differentiation of hNSCs into neurons by removal of growth factors, intracellular Ca^2+^ level immediately changes to activate certain signaling pathways. Therefore, intracellular Ca^2+^ levels were determined to evaluate the effect of TCQA at a very early phase of differentiation using Calcium KitⅡ-Fluo 4 (Dojindo, Japan).

hNSCs were seeded at 2.5 × 10^4^ cells/cm^2^ in black clear bottom 96-well plates (Greiner). After 48 h of incubation, cells were pre-treated with 100 μl of Loading buffer (5% Pluronic F-127,250 mmol/l Probenecid and 1 μg/μl Fluo 4 AM in Hanks’ HEPES Buffer) for 30 min. After that, the supernatant was removed and cells were washed with PBS and treated with differentiation medium without (control) or with 10 μM of TCQA. The cells were incubated at 37ºC for 1–180 min in the dark. After the treatment, fluorescence intensity (495 nm/535 nm) was detected using a microplate reader and was calculated as a percentage of control.

### Rhodamine 123 uptake measurement

Rhodamine 123 is a fluorescent probe that stains mitochondria directly. In this study, rhodamine 123 was used to evaluate mitochondrial membrane potential (ΔMMP). Once hNSCs were induced to differentiate, intracellular Ca^2+^ was released from the endoplasmic reticulum and subsequently, Ca^2+^ was transported to mitochondria. Because mitochondrial Ca^2+^ levels modulate ATP and ROS production, rhodamine 123 was used for evaluating mitochondrial activity. Rhodamine 123 (Wako, Japan) was dissolved in DMSO at 100 mg/ml. hNSCs were seeded at 2.5 × 10^4^ cells/cm^2^ in 96-well plates (BD Falcon) and incubated at 37ºC for 48 h. After the incubation, hNSCs were treated in a growth medium or a differentiation medium without (control) or with 10 μM of TCQA and were incubated at 37ºC for 30–180 min. After the treatment, hNSCs were washed with PBS and were treated with rhodamine 123 (10 μg/ml), dissolved in 10 mM HEPES-HBSS buffer (pH 7.4), and incubated at 37ºC for 20 min. After the incubation, hNSCs were washed with cold-PBS, lysed with 1% Triton-X solution (200 μl/well) and incubated at room temperature for 30 min avoiding light exposure. After the incubation, the cell lysate was transferred into a black clear bottom 96-well plate and the fluorescence intensity (507 nm/529 nm) was measured. Uptake levels of rhodamine 123 were calculated as a percentage of control.

### Measurement of intracellular ROS level

Removal of growth factors to induce differentiation of hNSCs causes an elevation of the intracellular Ca^2+^ level, which in turn activates ROS. Under normal physiological conditions, ROS activate certain signaling pathways, however, excess ROS can induce oxidative stress that harms cells. Therefore, the effects of TCQA on intracellular ROS level was determined using the OxiSelect Intracellular ROS assay kit (Cosmo Bio, Japan) following the manufacturer’s procedures.

hNSCs (2.5 × 10^4^ cells/cm^2^) were seeded in black clear bottom 96-well plates and were incubated in medium with TCQA for 1 h. The cells were then incubated with 10 μM DCFH-DA at 37°C for 1 h and washed with PBS. The fluorescence intensity of dichlorofluoresein (DCF) was measured using a microtiter plate (Dainippon Sumitomo Pharma Co, Ltd., Japan) reader at excitation wavelength of 485 nm and emission wavelength of 528 nm. Intracellular ROS was monitored using the fluorescent probe DCFH-DA. Intracellular H_2_O_2_ or low-molecular-weight peroxides oxidize DCFH-DA to the highly fluorescent compound DCF.

### RNA isolation from hNSCs

The hNSCs were seeded in 60-mm Petri dishes at the density of 2.5×10^4^ cells/cm^2^ and were incubated at 37ºC for 48 h. After that, cells were treated with differentiation medium with or without 10 μM of TCQA and were incubated at 37ºC for 6–24 h. After the treatment, cells were washed with ice-cold PBS. The total RNA was extracted from the cells using ISOGEN kit (Nippon Gene Co. Ltd., Toyama, Japan) following manufacturer’s instructions, as reported previously (Han et al., 2010). Total RNA was quantified and assessed for quality with a NanoDrop 2000 spectrophotometer (Thermo Scientific, Wilmington, DE, USA).

### DNA microarray analysis

Double-stranded cDNA was synthesized from 100 ng of total RNA with the GeneAtlas 3’ IVT Express Kit (Affymetrix Inc., Santa Clara, CA, USA). Biotin-labeled amplified RNA (aRNA) was synthesized by in vitro transcription using the GeneChip 3’ IVT Express Kit (Affymetrix Inc., Santa Clara, CA, USA). A total of 9.4 mg of purified aRNA was fragmented using the GeneAtlas 3’ IVT Express Kit and was hybridized for 16 h at 45°C using GeneChip MG-430 PM microarray (Affymetrix Inc., Santa Clara, CA, USA). The chip was washed and stained in the Gene Atlas Fluidics Station 400 (Affymetrix Inc., Santa Clara, CA, USA) and then the resulting image was scanned using the GeneAtlas Imaging Station (Affymetrix Inc., Santa Clara, CA, USA). Data analysis was performed using the Partek Express software (Partek Inc., St. Louis, MO, USA) provided by Affymetrix as part of their GeneAtlas system. Compared with the non-treated cells, fold change in expression in the TCQA-treated group was calculated and converted to log 2 data.

### Statistical analysis

All analyses were performed on blind-coded slides as previously described [47]. Statistical analysis was performed using GraphPad Prism 6 (GraphPad Software, Inc., San Diego, CA). A Student’s t-test was used when two groups were compared. Results are expressed as mean ± standard error of the mean (SEM). Statistical analysis of the results obtained in the MWM was carried out using two-way ANOVA with Ryan-Einot-Gabriel-Welsch multiple range test. One-way ANOVA followed by Ryan-Einot-Gabriel-Welsch multiple range test was also done. A P value of < 0.05 was considered statistically significant.

## SUPPLEMENTARY MATERIALS

Supplementary Table
