# Supplementary material for: 3,4,5-Tricaffeoylquinic acid induces adult neurogenesis and improves deficit of learning and memory in aging model senescence-accelerated prone 8 mice
Source: Aging (Albany NY). 2019 Jan 17;11(2):401–22. doi: 10.18632/aging.101748 (PMC6366991; doi:10.18632/aging.101748)
Supplement: Supplementary Table [file aging-11-101748-s001.pdf]

SUPPLEMENTARY MATERIAL

Supplementary Table 1. Expression changes of Actin cytoskeleton-related genes regulated by 3,4,5-triCaffeoylquinic acid (TCQA).

Gene title	Gene symbol	Fold change	Function	Category
EH Domain Binding Protein 1	EHBP1	1.63	actin binding, alternative splicing	Actin cytoskeleton
Protein Kinase N2	PKN2	1.40	inhibits Akt pro-survival-induced kinase activity, cell cycle progression, actin cytoskeleton assembly, cell migration, cell adhesion	
Radixin	RDX	1.40	regulation of actin polymerization and de- polymerization, regulation of actin length	
Transmembrane Protein 106B	TMEM106B	1.39	dendrite morphogenesis	
Kinectin 1	KTN1	1.37	microtubule-based process, microtubule-based movement, actin cytoskelton	
IQ Motif Containing GTPase Activating Protein 1	IQGAP1	1.36	actin cytoskeleton, calcium ion binding, binding activated cdc42, promote neurite outgrowth	
Actin, Alpha 2, Smooth Muscle, Aorta	ACTA2	1.35	actin cytoskeleton	
CDC42 Binding Protein Kinase Alpha	CDC42BPA	1.30	cytoskeleton organization, protein kinase activity, zinc ion binding	
Kelch Like Family Member 17	KLHL17	1.29	regulation of actin cytoskeleton organization, activation of protein kinase activity, this protein may play a key role in the regulation of actin-based neuronal function.	
Coiled-Coil Domain Containing 88A	CCDC88A	1.28	plays a role in cytoskeleton remodeling and cell migration, correct neuron positioning, dendritic development and synapse formation	
Junction Mediating And Regulatory Protein, P53 Cofactor	JMY	1.27	stem cell differentiation, acts both as a nuclear p53/TP53-cofactor and a cytoplasmic regulator of actin dynamics, may promote the rapid formation of a branched actin network	
LanC Like 2	LANCL2	1.25	cortical actin cytoskeleton	
transforming acidic coiled-coil-containing protein 1	TACC1	1.25	microtubule cytoskeleton, cell cycle	
Protein Phosphatase 1 Regulatory Subunit 12A	PPP1R12A	1.25	phosphoprotein phosphatase activity, ankyrin, actin cytoskelton	
Dynein Cytoplasmic 1 Light Intermediate Chain 2	DYNC1LI2	1.25	microtubule cytoskeleton, microtubule motor activity	
Dynein Cytoplasmic 1 Heavy Chain 1	DYNC1H1	1.23	microtubule cytoskeleton organization, cell cycle, microtubule motor activity	
Tropomyosin 4	TPM4	1.22	actin cytoskeleton	
